# Functional Analysis of the *GhIQD1* Gene in Cotton Resistance to Verticillium Wilt

**DOI:** 10.3390/plants13071005

**Published:** 2024-03-31

**Authors:** Jianglin Xu, Ting Zhou, Yongqiang Wang, Yejun Yang, Yuanchun Pu, Quanjia Chen, Kai Zheng, Guoqing Sun

**Affiliations:** 1 Engineering Research Centre of Cotton, Ministry of Education, College of Agriculture, Xinjiang Agricultural University, Urumqi 830052, China; 15334197038@163.com (J.X.); wcy694687@163.com (Y.W.); chqjia@126.com (Q.C.); 2 Biotechnology Research Institute, Chinese Academy of Agricultural Sciences, Beijing 100081, China; zz15034151584@163.com (T.Z.); 15903469823@163.com (Y.Y.); 3 College of Agronomy, Shanxi Agricultural University, Taigu, Jinzhong 030800, China; 4 Institute of Western Agriculture, The Chinese Academy of Agricultural Sciences, Changji 831100, China; puyuanchun1209@126.com

**Keywords:** cotton, chlorosis, plant hormones, calmodulin-binding protein

## Abstract

Cotton is a critical crop with massive economic implications worldwide. Verticillium wilt is a soil-borne ailment caused by *Verticillium dahliae*, which harms the growth and development of cotton. Therefore, investigating the genes associated with resistance to verticillium wilt is of particular significance. In this study, we identified the *GhIQD1* gene through transcriptome analysis and experimentally characterized the role of the *GhIQD1* gene in cotton against *V. dahliae*. The findings indicated that *GhIQD1* acts as a calmodulin-binding protein. The expression of *GhIQD1* was the highest in stems, and the expression level increased significantly following infection with *V. dahliae*. The expression in resistant cotton varieties was higher than in susceptible cotton varieties. Through overexpression of the *GhIQD1* gene in tobacco, these transgenic plants exhibited improved resistance to *V. dahliae*. In contrast, by silencing the *GhIQD1* gene in cotton through VIGS, the resistance to *V. dahliae* was reduced. Following inoculation, the leaves yellowed, and the disease index was higher. Transcriptome analysis of transgenic tobacco 72 h after inoculation indicated that overexpression of *GhIQD1* increased the enrichment of the calmodulin pathway and stimulated the production of plant hormones alongside secondary metabolites. Consequently, we investigated the relationship between the *GhIQD1* gene and plant disease-resistant hormones SA, JA, and ABA. In summary, this study uncovered the mechanism by which *GhIQD1* conferred resistance to *V. dahliae* in cotton through positive regulation of JA and ABA, providing crucial information for further research on the adaptation of plants to pathogen invasion.

## 1. Introduction

Cotton (*Gossypium* spp.) is a critical worldwide cash crop with diverse uses, making it a major source of income for farmers globally. However, cotton production faces a critical problem in the form of cotton verticillium wilt, caused by the *V. dahliae*, and is spread predominantly through soil. The incidence of verticillium wilt in cotton is very high, and the morbidity caused by the disease is also substantial. This has caused a huge loss of cotton yield and quality. Consequently, researchers have explored cotton verticillium wilt to identify effective control methods to limit the impact of the disease on cotton production and quality [1,2,3].

Plant immune regulation is an intricate and complex system. Ca^2+^ is an essential messenger for plant immune system functioning and establishes an extremely complex network composed of many interconnected nodes transmitting external or internal danger signals to activate various defense responses [4]. Recent studies point to the role of some Ca+ sensors in physiological processes related to immunity in developing plants [5,6,7]. AtCAMTA3 negatively regulates plant immunity after recognition of pathogen-associated molecular patterns (PAMPs), as well as non-host resistance to Mixanthomas [7]. CAMTA3 negatively regulates plant defense against biotrophic and necrotic pathogens by controlling endogenous levels of SA [8,9]. In addition, CAMTA3 has been proposed to control plant resistance to herbivorous insects by regulating glucosinolate metabolism [10,11]. The first calcium ion-independent CaM-binding motif identified was the IQ motif. The IQ motif-containing proteins in plants are the IQD protein family possessing the IQ67 domain, the IQM protein family, the cyclic nucleotide-gated channel (CNGC) protein family, and calmodulin binding transcriptional activators [12]. Cells employ intracellular calcium signaling to modulate functions through spatiotemporal specificity. This signaling can be caused by extracellular stimulants like pathogenic agents, plant hormones, and environmental stimuli such as alkali stress, salt, drought, and light, occurring with changes in intracellular calcium ion concentration.

We have identified the disease-resistant functional gene GhIQD1.IQD1 is a calmodulin protein and a member of the IQD family. There are many studies on IQD families. AtIQD1 acts in *Arabidopsis thaliana* as a positive regulator of glucosinolide (GS) accumulation and plant defense response to insects [13]. Five IQD genes were identified and analyzed in Chinese cabbage. *BrIQD67* transgenic plants increased drought resistance. The IQD (IQ35 domain) family has a critical role in various abiotic stress responses in plants [14]. Our study identified the driving-protein light chain associated protein-1 (KLCR 1) in Arabidopsis and demonstrated the association of IQD1 with microtubules [15]. Arabidopsis IQD16 protein has been identified as a microtubule-associated protein impacting cortical microtubule sequencing and cell expansion [16]. The IQD family also plays a vital role in fibrocyte development. *GhIQD21* is a *GhCaM7* interacting protein situated in MT (Micro tubules) that enables plant growth and potential cotton fiber development [17]. IQD genes can enhance drought tolerance, salt tolerance, and cold tolerance in cotton [18]. Studies have demonstrated that IQ domain proteins play regulatory roles in plant growth and adaptation to environmental alterations.

A plant’s first line of defense is activated by interaction either between plasma membrane bound pattern-recognition receptors (PRRs) with several elicitors, such as PAMPs. PRR initiates an overall defense response through activating PAMP-triggered immunity (PTI), followed by rapid activation of MAPKs, oxidative burst, and Ca^2+^ influx at the plasma membrane [19] and finally augmenting biosynthesis of plant defense hormones, namely, salicylic acid (SA), jasmonic acid (JA), and their derivatives [20]. Microbe-induced transient increase of Ca^2+^ flux is the most important upstream signaling event leading to SA biosynthesis. SA is a crucial plant defense-related hormone that influences systemically acquired resistance and broad-spectrum persistent disease resistance [21,22,23]. JA is crucial for plant defense responses to necrotrophic and biotrophic fungal infections [24]. These two pathways are predominantly adversarial, and the balance of crosstalk between them influences the pathology. Studies have demonstrated that the IQD family is involved in plant hormone regulation. The bamboo IQD gene family exhibited a tissue-specific pattern and was involved in methyl jasmonate and drought stress [25]. The soybean IQD gene family also specifically expresses and regulates methyl jasmonate stress [26].

Upland cotton is the largest cultivated variety in China, playing a crucial role in supporting the country’s economic development. However, the invasion of *Verticillium dahliae* has inflicted significant losses on cotton farmers. Due to the lack of chemical agents for controlling cotton verticillium wilt, farmers can only resort to cultivation measures such as crop rotation, intercropping, and deep tillage to mitigate its impact. Research on disease-resistant genes holds significant importance for breeding disease-resistant cotton varieties. We have identified the disease-resistant functional gene GhIQD1 The IQD1 overexpression line (IQD_1OXP) also has strong resistance to necrosis boea mold [27]. IQD1 and related proteins provide scaffolds enabling RNA transport along microtubules, controlling and fine-tuning gene expression and protein sorting, explaining the pleiotropic role of IQD1 in many cellular pathways [28]. We employed VIGS to silence GhIQD1 and ascertain its contribution to resistance against Verticillium dahliae in cotton. The study investigated the variations of SA, JA, and ABA (Abscisic acid) in the expression of the cotton verticillium wilt resistance gene GhIQD1 and their roles in its expression. This research provides a theoretical foundation for studying the signaling transduction pathways and molecular mechanisms involved in cotton’s resistance to verticillium wilt, as well as for the breeding of highly resistant varieties.

## 2. Results

### 2.1. GhIQD1 Sequence Analysis

**Figure 1:**
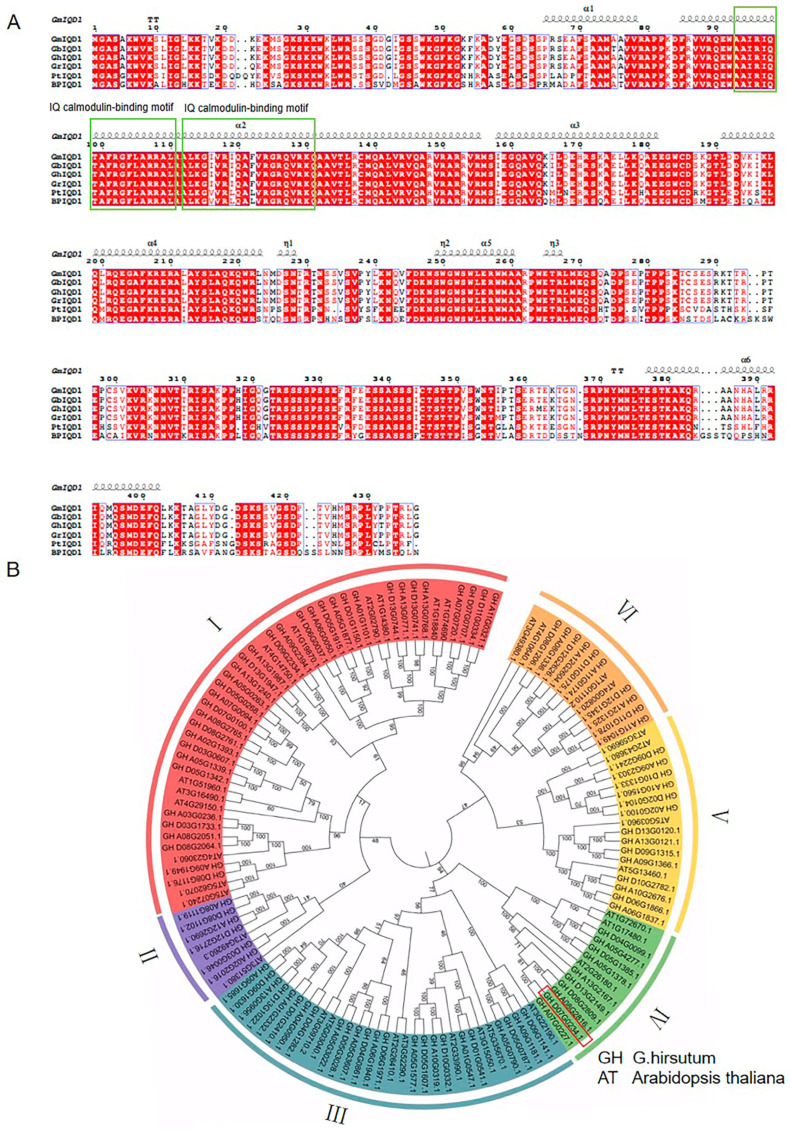
Conservation and evolutionary relationship of *GhIQD1* (**A**) *GhIQD1* multiple sequence alignment (**B**) *GhIQD1* phylogenetic tree the evolutionary tree is divided into I to VI taxa.

The early stage of the laboratory. Through transcriptome analysis 72 h after inoculation in cotton of susceptible and resistant varieties at squaring stage, genes *GhIQD1* were obtained, and their expression levels were significantly increased in resistant varieties after infection with *V. dahlia* [29]. The CDS length of the calmodulin-binding protein *GhIQD1* gene derived from the resistant variety Zhimian 2 (ZM2) of upland cotton is 1314 bp, encoding 437 amino acids. The total number of negatively charged residues (Asp + Glu) is 41, while the number of positively charged residues (Arg + Lys) is 82. The isoelectric pI of the protein is 10.48. The instability coefficient of the protein is 53.68. Two conserved functional structural sites for the IQ calmodulin-binding motif were identified in *GhIQD1*. Multi-amino acid sequence alignment showed that *GhIQD1* was highly conserved across different cotton genera (Figure 1A), demonstrating high consistency with the *IQD1* of poplar and mulberry. A phylogenetic tree was constructed with 99 IQD proteins and 33 *Arabidopsis thaliana* from upland cotton [12]. Phylogenetic analysis showed that this gene had increased homology with the *Arabidopsis* AT2G26180 gene, with both belonging to Group IV (Figure 1B), indicating that they may have similar functions.

### 2.2. Analysis of GhIQD1 Expression Patterns

**Figure 2:**
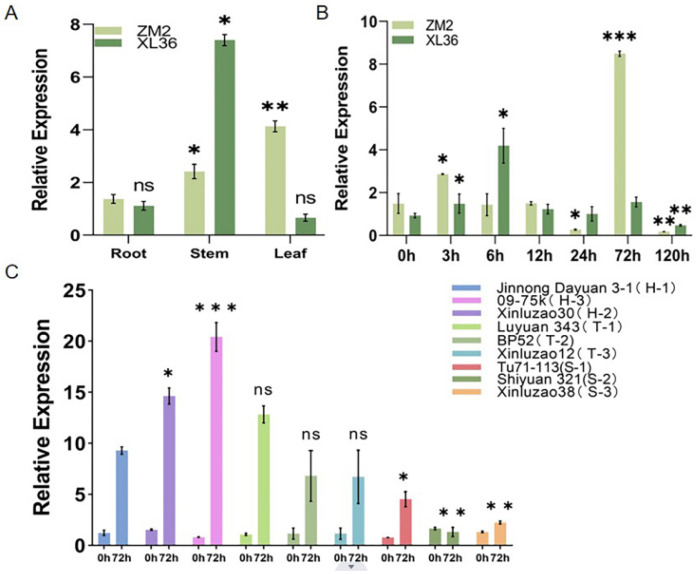
Expression characteristics of *GhIQD1*. (**A**) Expression levels of *GhIQD1* in the roots, stem, and leaves of cotton. (**B**) Expression levels of *GhIQD1* at 0 h, 3 h, 6 h, 12 h, 24 h, 72 h, and 120 h after infection with *V. dahliae*. *** *p* < 0.001; Student’s *t*-test. (**C**) Expression levels of *GhIQD1* in various resistant varieties. Values are the mean and standard deviation (SD). Error bars represent the SD. * *p* < 0.05; ** *p* < 0.01; *** *p* < 0.001; ns: No statistical significance Student’s *t*-test. All experiments were repeated in at least triplicate.

The expression levels of *GhIQD1* in the root, stem, and leaf tissues of ZM2 and Xinluzao 36 (XL36) indicated that *GhIQD1* was expressed throughout all cotton tissues, with ZM2 being higher than XL36 in root and leaf tissues. However, XL36 exhibited higher expression than ZM2 in the leaves (Figure 2A). The expression pattern of *GhIQD1* when exposed to verticillium wilt was analyzed. The results indicated that the gene expression level increased after 3 h of inoculation with *V. dahliae* compared to the control group. The *GhIQD1* expression level was significantly upregulated after 72 h of inoculation with *V. dahliae* (Figure 2B). To verify the stable expression in resistant varieties. Therefore, we chose three high-resistance varieties, namely Jinnong Dayuan 3-1 (H-1), Xinluzao30 (H-2), and 09-75k (H-3), alongside three disease-resistant varieties, namely 343 (T-1), BP52 (T-2), and Xinluzao12 (T-3), and three susceptible varieties, Tu71-113 (S-1), Shiyuan 321 (S-2), and Xinluzao38 (S-3). To confirm the expression characteristics of the *GhIQD1* gene in different responses after inoculation, we found that the expression of *GhIQD1* was significantly higher in disease-resistant varieties than in susceptible varieties (Figure 2C). These findings indicated that the *GhIQD1* gene significantly responded to *V. dahliae* in resistant varieties.

### 2.3. Construction and Silencing Efficiency Examination of the VIGS Vector of the GhIQD1 Gene

**Figure 3:**
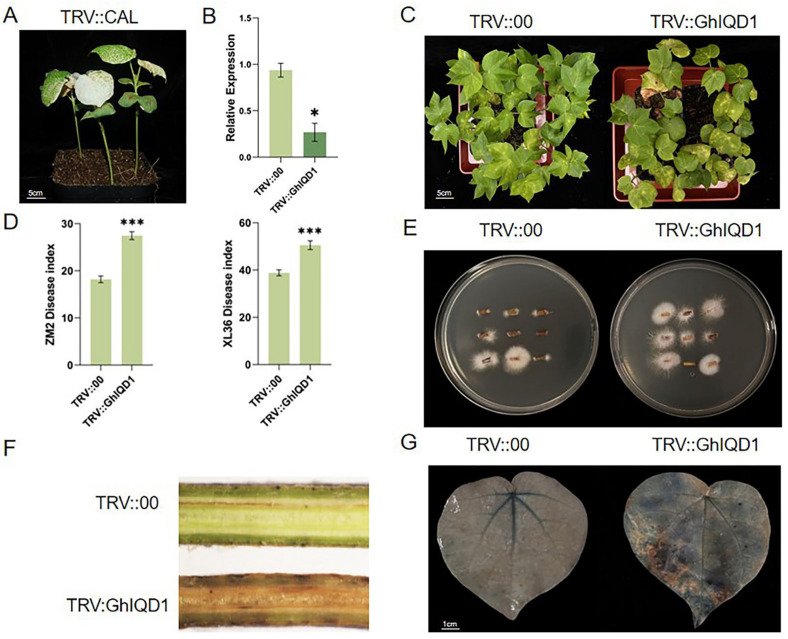
Silencing of *GhIQD1* in cotton limits plant resistance to *V. dahliae*. (**A**) Albino phenotype of TRV::GhCLA 15 days following VIGS. (**B**) The silencing efficiency of *GhIQD1* was measured by RT-qPCR. (**C**) Verticillium wilt phenotypes in TRV::00 and TRV::GhIQD1 plants after 25 days of inoculation; (**D**) disease index of different varieties at 25 days; (**E**) isolation of *V. dahliae;* (**F**) stem profiling; (**G**) staining of the first true leaf with Trypan blue. Values are the mean and standard deviation (SD). Error bars represent the SD. * *p* < 0.05; *** *p* < 0.001; Student’s *t*-test. All experiments were repeated in at least triplicate.

To examine the role of the *GhIQD1* gene in cotton resistance to *V. dahliae*, it was silenced using VIGS. Labeled vectors (TRV::CLA) and empty vectors (TRV::00) were employed as positive and negative controls, respectively. Phenotypes in albino TRV::CLA plants appear phenotypic (Figure 3A). The results indicate that the VIGS system can work normally in upland cotton. *GhIQD1* levels were measured using RT-qPCR, demonstrating effective silencing in TRV::GhIQD1 cotton (Figure 3B). Silenced *GhIQD1* cotton and control cotton were inoculated with Vd592. The findings indicated that the leaves were yellowed and wilted. (Figure 3C). This was more evident in TRV::GhIQD1 plants compared to the control TRV::00 plants. Disease index of TRV::GhIQD1 was higher in both susceptible and resistant varieties than the control plants (Figure 3D). Similarly, fungal recovery experiments demonstrated that fungal biomass colonization was more widespread in TRV::GhIQD1 (Figure 3E). Compared to TRV::00, the area of cell death in the cotton leaves of TRV::GhIQD1 was more expansive (Figure 3G). The TRV::GhIQD1 plant cotton stem segment longitudinal browning degree is also higher (Figure 3F). These findings suggest that the silencing of TRV::GhIQD1 reduces the resistance of upland cotton to *V. dahliae*.

### 2.4. Overexpression of GhIQD1 Improved the Resistance of Tobacco to V. dahliae

**Figure 4:**
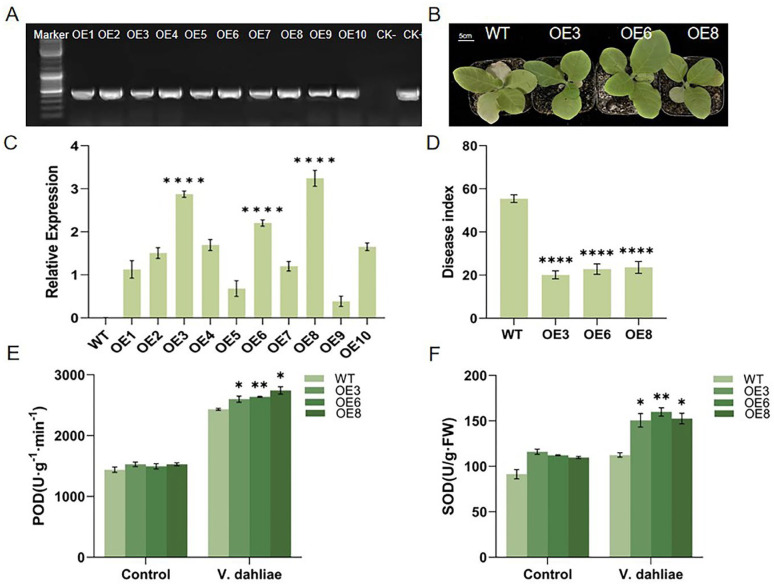
Overexpression of *GhIQD1* increased the resistance of tobacco to *V. dahliae*. At 20 days of age, tobacco was inoculated with Vd592. (**A**) Transgenic lines were tested using PCR; (**B**) *GhIQD1* expression levels in transgenic lines and wild types were examined by RT-qPCR; (**C**) phenotypic map of transgenic tobacco 14 days after inoculation; (**D**) disease index 18 days after inoculation; (**E**) POD activity and (**F**) SOD activity. Values are the mean and standard deviation (SD). Error bars represent the SD. * *p* < 0.05; ** *p* < 0.01; **** *p* < 0.0001; Student’s *t*-test. All experiments were repeated in at least triplicate.

To examine the function of *GhIQD1*, the leaf disk method was used to transfect the *GhIQD1* gene into tobacco. A total of ten tobacco T0 generation plants were obtained (Figure 4A). RT-qPCR was conducted on the *GhIQD1* gene in ten transgenic tobacco lines, and OE3, OE6, and OE8 with stable expression of *GhIQD1* and high expression were selected to reproduce the T2 generation (Figure 4B). Tobacco seedlings of WT, OE3, OE6, and OE8 at seedling ages of 20 days were soaked in a Vd592 spore suspension at a concentration of 1 × 10^7^/mL. Following 14 days of treatment, the symptoms of wild-type tobacco leaf necrosis and yellowing were significantly reduced compared to transgenic leaves (Figure 4C), and the disease index of wild-type tobacco plants was significantly lower than wild-type tobacco plants 18 days after inoculation (Figure 4D). These findings suggest that overexpression of *GhIQD1* increases resistance to *V. dahliae* in tobacco.

Prior studies have demonstrated that SOD (Superoxide dismutase) activity increases upon an increased amount of superoxide free radicals in the body when plants are susceptible to disease [30]. POD (Peroxidase) participates in various biological and physiological processes, using H_2_ O_2_ as a substrate for oxidation. In this study, to confirm if the enhancement of disease resistance of *GhIQD1* transgenic tobacco is related to the endogenous SOD and POD activities of *GhIQD1*, we examined the endogenous SOD and POD in transgenic tobacco. When inoculated with *V. dahliae* for 48 h, all *GhIQD1* transgenic strains exhibited significantly higher SOD and POD levels compared to control plants (Figure 4E,F).

### 2.5. Transcriptome Analysis of the Resistance Mechanism of GhIQD1

**Figure 5:**
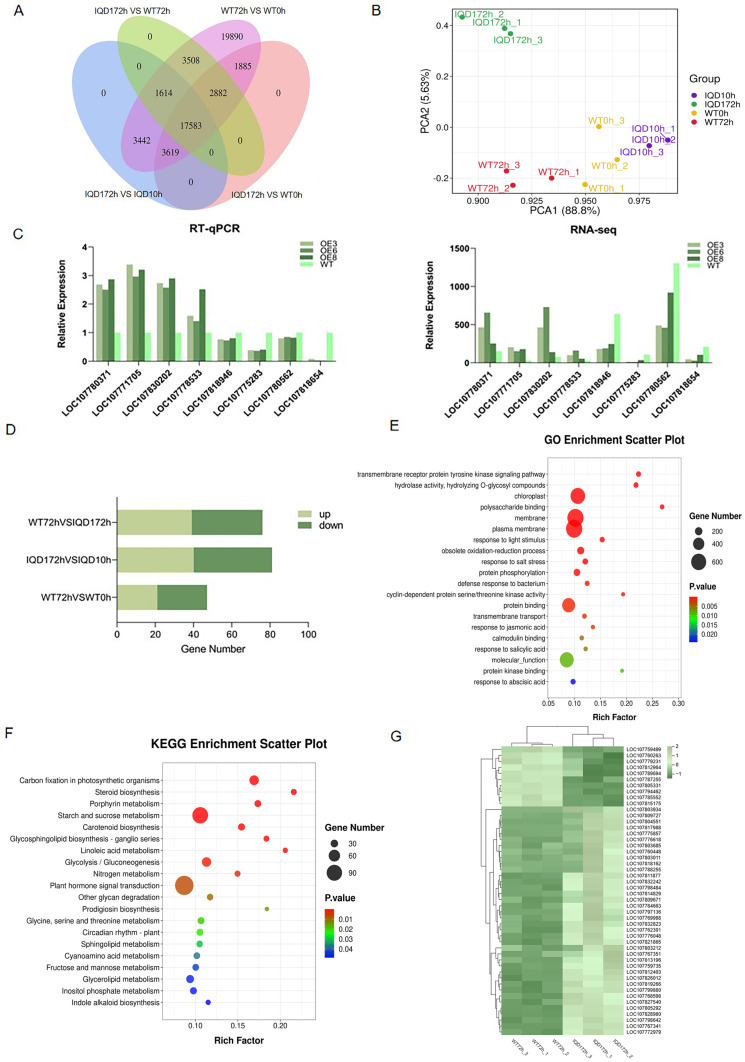
Transcriptomic analysis. (**A**) Venn diagram of differential genes; (**B**) PCA diagram; (**C**) differential gene verification; (**D**) calmodulin binding differential gene; (**E**) GO enrichment map; (**F**) KEGG enrichment map; (**G**) expression analysis of calmodulin binding related genes in the transcriptome.

To identify the resistance mechanism of the *GhIQD1* gene following infection, we conducted full transcriptomic sequencing (RNA-seq) analysis for 72 h after infection of overexpressing tobacco with *V. dahliae*. PCA (Figure 5B) investigation exhibited good clustering between the wild-type (WT) and overexpressing (OE) datasets. In this analysis, 25,969 genes were upregulated, and 26,438 genes were downregulated in OE3. In OE6, 26,204 genes were upregulated, and 26,258 genes were downregulated. In OE8, 27,070 genes were upregulated, and 25,587 genes were downregulated. In WT, 25,989 genes were down-regulated and 25,691 genes were up-regulated. We generated Venn diagrams of differential genes (Figure 5A) and identified more differentially expressed genes in overexpressed tobacco, suggesting that a large-scale transcriptome reprogramming event took place in tobacco after inoculation with *V. dahliae*. To characterize the accuracy of the transcriptome data, we randomly selected four upregulated (LOC107780371, LOC107771705, LOC107830202, and LOC107778533) and four downregulated (LOC107818946, LOC107775283, LOC107780562, and LOC107818654) transcriptome DEGs for RT-qPCR analysis. The findings demonstrated that the expression trends of the two groups of genes were consistent, confirming the accuracy of our transcriptome results (Figure 5C).

Difference multiple FC ≥ 2 or FC ≤ 0.5 and q value < 0.05 (|log2fc| ≥ 1&q < 0.05) were used as the threshold criteria, and WT and OE were selected 72 h after inoculation for GO and KEGG enrichment analysis. Thus, compare what disease-resistance pathways are stimulated by overexpression of tobacco. Graphene oxide enrichment analysis results showed (Figure 5E) that these DEGs were predominantly involved in the signaling pathway of intracellular receptor protein tyrosine kinase. This signaling pathway is a bridge between plant cells and the external environment, regulating plant responses to pathogen infection and responses to light stimulation. Redox enzyme activity regulates redox processes in the cell and helps maintain the redox balance. Moreover, the response to salt stress, protein serine/threonine kinase activity, defense response against bacteria, transmembrane transport response, ABA (abscisic acid) response, calmodulin binding, SA response, signal transduction, and JA response are highly linked to plant immune response. Additionally, enrichment in the polysaccharide binding pathway was observed. Plant polysaccharides have specific antibacterial activity, and can act in signal transduction networks of plants, triggering a series of signaling pathways for disease resistance. This signaling assists plants in responding quickly and precisely to pathogen attacks.

KEGG enrichment analysis demonstrated (Figure 5F) that the metabolic pathways of photosynthesis, glycine and dicarboxylic acid metabolism, porphyrin metabolism, and starch and sucrose metabolism played a critical role, providing the synthesis pathway of organic matter and energy generation. These pathways were followed by plant hormone signaling, indole alkaloid biosynthesis, glycine, serine, and threonine pathways, phenylalanine, tyrosine, and tryptophan biosynthesis, as well as alanine, aspartate, and glutamate metabolism. These amino acids act as precursors of bioactive compounds, aromatic amino acids, and other amino acids, improving plant resistance to oxidative stress by synthesizing antioxidants, including polyphenols and flavonoids, and can also form precursors to secondary metabolites, including disease-fighting compounds and alkaloids in plants. Plant hormones have a critical role in plant disease resistance. They can regulate various growth and development processes, and are involved in plant disease resistance and defense mechanisms [31]. These plants were also enriched in cyanuric acid metabolism, fructose metabolism, and hydrated inositol phosphate metabolism. Cyanuric acid is an antioxidant capable of improving plant resistance to fungi by inhibiting fungal growth and is involved in signaling pathways that can activate plant defense systems and enhance resistance to pathogens. Accumulation of inositol phosphate under stress exposure, including pathogen invasion, may improve plant adaptability to stress. These metabolic pathways interact with each other to form complex regulatory networks taking part in plant disease resistance response. Examining the role of these metabolic pathways in plant disease resistance is helpful in understanding the defense mechanisms of plants and provides a scientific basis for breeding resistant varieties and developing plant protection strategies.

Based on the results of GO and KEGG enrichment, the overexpression of *GhIQD1* in tobacco can promote the production of photosynthetic substances, enrich a large number of amino acid metabolic pathways, and stimulate plant secondary metabolic synthesis pathways, plant hormone responses, and extensive transmembrane transport reactions. These impacts may directly or indirectly influence the resistance of cotton to pathogens. Prior studies have demonstrated that calmodulin binding is associated with the resistance and defense response of plants [32]. A comparison of WT0h vs. WT72h, IQD10h vs. *IQD172h*, and IQD172h vs. WT72h demonstrated that calmodulin binding resulted in more extensive genetic changes in OE plants (Figure 5D). The majority of these genes were upregulated (Figure 5G). In response to the enrichment of ABA in GO, genes linked to ABA pathways in OE plants resulted in more extensive changes than in WT. Overall, transcriptomic results from infection with *V. dahliae* showed that OE plants had greater growth and physiological changes with more rapid and intense responses. These results provide a reference for further study of plant adaptation mechanism to verticillium wilt.

### 2.6. The GhIQD1 Gene Is Associated with the Regulation of JA, ABA, and SA Signaling Pathways

**Figure 6:**
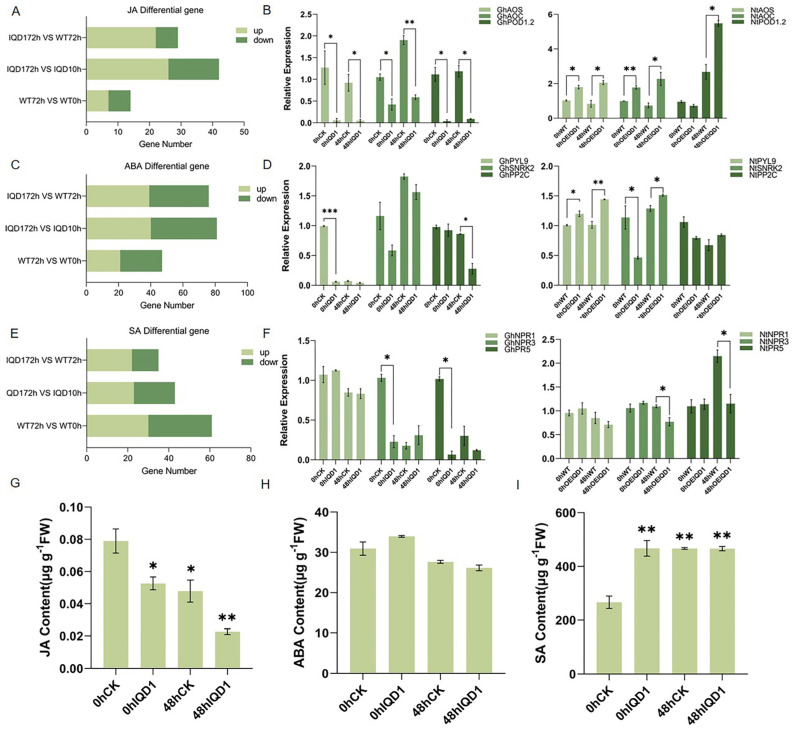
Impacts of *GhIQD1* on JA, ABA, and SA signaling pathways. (**A**) JA differentially expressed genes in the transcriptome; (**B**) JA pathway marker genes in cotton and tobacco after infection with *V. dahliae*; (**C**) ABA differentially expressed genes in transcriptomes; (**D**) ABA pathway marker genes in cotton and tobacco after infection with *V*. *dahlia;* (**E**) SA differentially expressed genes in transcriptomes; (**F**) SA pathway marker genes in cotton and tobacco after infection with *V. dahliae*; (**G**) JA content in leaves of TRV::00 plants and TRV::GhIQD1 plants 48 h after *V. dahliae* exposure; (**H**) ABA content in leaves of TRV::00 plants and TRV::GhIQD1 plants 48 h after *V. dahliae* exposure; (**I**) SA content in leaves of TRV::00 plants and TRV::GhIQD1 plants 48 h after *V. dahliae* exposure; Values are the mean and standard deviation (SD). Error bars represent the SD. * *p* < 0.05; ** *p* < 0.01; *** *p* < 0.001; Student’s *t*-test. All experiments were repeated in at least triplicate.

Hormones are essential for regulating responses to diverse adversities [33,34]. SA- and JA-mediated signaling pathways are involved in plant immune responses, and IQD family members mediate the regulation of SA and JA. *GhIQD1* may be involved in disease resistance associated with hormone response. Based on transcriptomic enrichment pathways of *GhIQD1*, it was found that it may be involved in ABA signaling pathways. Comparing the transcriptome data of WT0h vs. WT72h, IQD10h vs. IQD172h, and IQD172h vs. WT72h, we determined that JA and ABA pathways resulted in more extensive gene changes in OE plants. Many of the genes were upregulated (Figure 6AC). However, the SA pathway did not change markedly (Figure 6E). To delve deeper into the role of *GhIQD1* in cotton resistance to verticillium wilt, we identified several marker genes associated with JA, ABA, and SA pathways in TRV::GhIQD1 cotton. Following inoculation, the expression of JA-related *AOC* (Allene oxide cyclase) genes in TRV::GhIQD1 cotton was significantly decreased compared to TRV::00 cotton. The expression of *AOS* and *PDF1.2* genes in TRV::GhIQD1 cotton was also significantly reduced compared to TRV::00 cotton (Figure 6B). These genes are upregulated in overexpressed tobacco (Figure 6B). We found that *PYL9*, *SnRK2*, and *PP2C* in ABA-associated pathways were downregulated in silenced plants infected with verticillium wilt. Compared to WT, *PYL9*, and *SnRK2* genes were upregulated in overexpressed tobacco plants, while *PP2C* was slightly upregulated (Figure 6D). The expression of the SA-related gene *NPR1* in TRV::GhIQD1 cotton was slightly decreased compared to TRV::00 cotton. *NPR3* in TRV::GhIQD1 cotton was higher than in TRV::00 cotton. *PR5* decreased significantly from TRV::00 cotton (Figure 6F). These results indicated that *GhIQD1* may influence the resistance of cotton to verticillium wilt by regulating JA, ABA, and SA signaling pathways. To further validate the expression results of the marker genes and assess the function of *GhIQD1* in cotton, we measured JA, ABA, and SA following the inoculation of plants with *V. dahliae*. The expression of marker genes for JA, ABA, and SA signaling pathways was consistent with that following *V. dahliae* treatment, and the SA content in TRV::GhIQD1 cotton was essentially the same as in TRV::00 cotton after treatment for 48 h (Figure 6I). However, JA and ABA contents in TRV::GhIQD1 cotton were significantly lower than in TRV::00 cotton (Figure 6G,H). Hormone pathway regulation is a complex network, and *GhIQD1* may impact JA and ABA-related pathways to confer verticillium wilt resistance.

## 3. Discussion

Ca^2+^ has a crucial role in plant innate immunity, and Ca^2+^ influx is an early event in plant resistance to pathogen attacks [35,36]. Ca^2+^ tags are decoded by diverse Ca^2+^ binding proteins, including CaM, which regulates various cellular processes, including gene regulation, protein synthesis, and ion homeostasis [37]. In eukaryotic cells, sensory environmental changes and stresses result in cytoplasmic Ca^2+^ increases. The accumulation of Ca^2+^ induced by *V. dahliae* promotes the acetylation of calmodulin *GhCaM7*, activating JA and ROS defense signaling pathways and changing cellular osmotic potential to enhance the resistance of cotton to verticillium wilt [38]. In this study, we determined that the disease resistance of cotton following silencing of *GhIQD1* was reduced compared to normal plants. The gene *GhIQD1* was overexpressed in the model tobacco plants, and the transgenic tobacco plants had significantly increased disease resistance compared to the wild-type. This makes *GhIQD1* applicable for genetic engineering to enhance the resistance of cotton to verticillium wilt.

microRNA silencing of viral gene expression and the accompanying salicylic acid-mediated defense response is the first line of defense [39], playing an essential role in the innate immune response of plants to pathogen infection [40,41,42]. The interaction between CaM1, CaM4, or CaM 7 and JAV1 depended on an increase in Ca^2+^ levels at the time of injury, inducing phosphorylation of JAV1. This results in relieved JA biosynthesis inhibition, leading to a rapid accumulation of JA biosynthesis, triggering plant defense response [43]. Prior studies have shown that *IQD1* overexpression lines (*IQD1* OXP) are more resistant to the necrosis-type fungus *Botryosphaeria*, while *IQD1* knockout lines (iqd 1-1) are more sensitive, with *IQD1* upregulated by jasmonic acid (JA) and downregulated by salicylic acid (SA) [27]. Additionally, overexpression of *IQD1* reduces the phytophagy of insects [13]. *GhCPK 33*, a Ca^2 +^-dependent protein kinase that directly modulates JA biosynthesis, is a negative regulator of the resistance to *V. dahliae* [44]. Exogenous application of JA-induced cytoplasmic free Ca^2+^ in *Arabidopsis* leaves [45] resulted in rapid upregulation of *GhIQD1* expression, while the application of SA had little effect, and JA level was significantly limited in TRV::GhIQD1 infected with *V. dahliae*. In TRV::GhIQD1, downregulated genes related to the JA signaling pathway were observed. These results indicate that the JA signaling pathway is related to *GhIQD1*-mediated disease resistance, and the JA signaling pathway and Ca^2+^ signaling pathway are related to plant disease resistance. Abscisic acid (ABA) is a key hormone for plant stress responses, growth, and development, which can promote plant drought tolerance and disease tolerance while inhibiting seed germination [46,47]. Crosstalk between Ca^2+^ and ABA signaling is well-established in regulating stomatal openings. These two pathways positively regulate one another [48]. Previous studies have demonstrated that IQD family members are associated with ABA regulation. The tomato IQD gene *SUN24* regulates seed germination through the ABA signaling pathway [49]. During seed germination, Ca^2+^ signaling negatively regulated the ABA signal. Salt hypersensitive (SOS 3)-like calcium-binding protein 5 (SCaBP 5) and Ca^2+^ dependent protein kinase 12 (CPK 12) operate as negative regulators of ABA signaling during Arabidopsis seed germination and early seedling growth [50,51]. It was found that ABA-related genes were downregulated in TRV::GhIQD1 plants, but upregulated in transgenic tobacco.

We characterized the calmodulin-binding protein gene *GhIQD1* as a positive regulator in response to *V. dahliae* infection in cotton and tobacco. *IQD1* can respond to pathogen infection, interact with other proteins, and regulate the expression of plant disease resistance genes, improving plant disease resistance. IQD family members can also be necessary to respond to other plant stresses. For instance, knockdown of *GhIQD31* and *GhIQD32* can elevate cotton sensitivity to drought and salt stress [18]. The ectopic expression of *BrIQD35* promoted tolerance of tobacco to drought stress [52]. The stress resistance of plants was increased by regulating ionic balance, protecting plant cell membranes, and inhibiting oxidative stress. *GhIQD1* is a candidate gene that can be employed to further understand the molecular mechanism of cotton resistance to verticillium wilt and to develop new germplasm resources.

## 4. Materials and Methods

### 4.1. Plant Growth Conditions and Culture of Test Strains

ZM2 and XL36 seeds were selected from control materials with full grain resistance, and nine cotton varieties were chosen from the conventional varieties containing 63 materials. Three highly resistant varieties, three disease-tolerant varieties, and three susceptible varieties were soaked until exposure to light, and the varieties were planted in a culture medium composed of vermiculite and nutrient soil (peat soil)1:2. Each tray contained ten 10 × 10 seedling boxes, each containing three to four seeds. Under the conditions of 25 °C, a cycle of 16 h of light/8 h of darkness, a light intensity of 120 μmol m^−2^s^−1^, and relative humidity maintained at around 60%, VIGS injection was conducted when the cotton seedling had a fully unfolded cotyledon. When the true leaves of the cotton seedling were unfolded, the root, stem, and leaf tissues were selected as test materials, and three groups of biological repeats were obtained from each sample and frozen at −80 °C for future tissue expression characteristic analysis. Cotton seedlings with consistent growth conditions were selected for inoculation. Wild-type tobacco and genetically modified tobacco (OE3, OE6, OE8) were grown at a constant temperature of 30 °C using a 16 h light/8 h darkness cycle.

The examined strain was *Verticillium dahliae* Vd592 (CGMCC preservation number: 3.3758), operating as the dominant strain of verticillium wilt of cotton in Xinjiang, belonging to the deciduous strain with robust pathogenicity. Verticillium Vd592 was stored at −80 °C, and was then streaked on PDA medium and cultured at 25 °C for four to five days. Single colonies were selected and cultured in Charlton’s liquid medium at 25 °C with shaking at 220 rpm for three to four days in the dark. The medium was then strained using four layers of gauze. The cultured solution was utilized to count the number of spores using a hemacytometer. Finally, the *V. dahliae* spores were suspended using sterilized ddH_2_O to a final concentration of 1.0 × 10^7^ per mL for inoculation [53]. The roots of cleaned cotton seedlings were incubated in the Vd592 spore suspension, and sterilized ddH_2_O was employed as the control treatment. Root tissues from biological triplicate cotton plants were obtained at different time points. The materials were flash-frozen in liquid nitrogen for expression analysis following inoculation.

### 4.2. Cloning and Sequence Analysis of the GhIQD1 Gene

The full-length sequence of gene GH_D07G0234 was obtained from the CottonFGD website. It was highly homologous to the *Arabidopsis* gene *ATIQD1* and was therefore named *GhIQD1*. Its sequence was amplified from the cDNA of ZM2 using Primer Premier 5. The clones were inserted into a 5xTA/Blunt Zero Cloning Mix vector and transformed into *E. coli* DH5α receptor cells using a heat shock approach. The monoclones were selected for PCR-positive identification, and the correct clones were chosen for sequencing in Sendangong Bioengineering (Shanghai, China) Co., Ltd.

Protein sequences were identified using BLAST (http://www.example.com (accessed on 1 December 2023)) according to the *GhIQD1* validation sequence. *IQD1* sequences were downloaded for other species from NCBI (https://www.ncbi.nlm.nih.gov/ (accessed on 12 December 2023)). The online software ESPript 3 (https://espript.ibcp.fr/ESPript/ESPript/ (accessed on 12 December 2023)) was used to conduct multiple sequence alignment analysis, alongside MEGA11 software modeling for neighbor-joining construction of an *IQD1* gene phylogenetic tree. The ExPASy program (http://www.expasy.org/ (accessed on 12 December 2023)) was employed to calculate protein parameters, including molecular weight, theoretical isoelectric point (pI), and amino acid composition.

### 4.3. Analysis of GhIQD1 Expression Patterns

The expression levels of *GhIQD1* in the root, stem, and leaf tissues of ZM2 and XL36 were analyzed at two leaf stages. The root systems of at least five plants from each treatment were obtained at 0, 3, 6, 12, 24, 72, and 120 h following inoculation and flash-frozen using liquid nitrogen. Total RNA was extracted from cotton seedlings utilizing a FastPure Plant Total RNA Isolation Kit (China Nanjing Novizan Biotechnology Co., Ltd., Nanjing, China). The first strand cDNA was synthesized using a PrimeScript™ RT kit (TaKaRa, Dalian, China) according to the manufacturer’s instructions. ChamQ Universal SYBR qPCR Master Mix (Vazyme, Nanjing, China) was used on an ABI 7500 real-time PCR system (Applied Biosystems, Foster City, CA, USA) for RT-qPCR. *GhUBQ7* served as the internal reference gene, and 2^−ΔΔCt^ was used to determine the relative expression level of the target gene [54]. The RT-qPCR assay was conducted using three biological replicates.

### 4.4. A VIGS Vector Was Constructed for Infection, and Trypan Blue Staining

According to the open reading frame sequence of the *GhIQD1* gene, the specific amplified fragment was 500 bp. The *Eco*R1 and *Bam*H1 cleavage sites were introduced into the 5′ end of the upstream and downstream primers, and the gene was cloned into *Escherichia coli* for use as the template. The target fragment was obtained by PCR amplification and inserted into the recombinant plasmid and the empty plasmid of tobacco crackling virus pTRV2. The target fragment and the linearized pTRV2 plasmid were recovered through double enzyme digestion with *Eco*RI and *Bam*H1. The target fragment was inserted into the linearized plasmid using seamless ligase from Vazyme (Nanjing, China). The expression vector pTRV2:GhIQD1 was constructed and transformed into *Escherichia coli*, which was verified by double enzyme digestion. A sample of 10 μL of verified recombinant plasmid was used to transform *Agrobacterium* “GV3101” through the freeze–thaw method, and monoclones were selected for colony PCR characterization. The identified and correct bacteria were further propagated, and 70% glycerol was added for long-term storage at −80 °C. The cotton seedlings, grown to the two-cotyledon stage, were injected with *Agrobacterium*. Plants infected with TRV::GhIQD1, TRV::00, and TRV::GhCLA were treated as the experimental group, negative control, and positive control, respectively. Approximately 15 days after infection, when the leaves of positive control plants exhibited an albino phenotype, samples were obtained from the second true leaves and roots of the experimental group, as well as negative and positive controls, with three replicates taken from each sample. RT-qPCR was employed to detect the silencing efficiency of genes.

The leaves were immersed in 5 mL of Trypan Blue staining solution (250 μg mL^−1^). Trypan Blue 25% (*w*/*v*) lactic acid, 25% water-saturated phenol, 25% glycerin, H_2_O, allowed to vacuum infiltrate for 5 min, and permeate for 5 min. The sample was then heated in boiling water for 2 min and cooled, then chloral hydrate solution (25 g dissolved in 10 mL of water) was used for decolorization. After multiple exchanges of chloral hydrate solutions, the sample was equilibrated in 70% glycerin for several hours [55].

### 4.5. Transgenic Tobacco

To explore the role of the *GhIQD1* gene in plant resistance to verticillium wilt, the plant expression vector CaMV35s:GhIQD1 was developed to transform tobacco. The specific primer fragment was 1314 bp based on the CDS sequence of the *GhIQD1* gene. The pCAMBIA2300 plasmid was used to transform tobacco by an *Agrobacterium*-mediated approach for specific reference.

### 4.6. Determination of SOD and POD Activity in Transgenic Tobacco

The infected leaves were collected 48 h after inoculation from tobacco seedlings with a seedling age of 20 days to determine POD content and SOD activity. Under normal or inoculated conditions, the total SOD activity of the purified protein and endogenous SOD activity of the sample was determined through the WST-8 method. Commercial bovine erythrocyte Cu/ZnSOD was utilized as a positive control (Acmec Biochemical, Shanghai, China). After weighing and grinding in liquid nitrogen, 0.3 g of the sample was placed in a 15 mL centrifuge tube, adding 6 mL of pre-cooled phosphate buffer (pH 7.8), and allowing to leach at 4 °C for 12 h (requiring frequent shaking), centrifuging at a low temperature at 10,000 rpm for 20 min to obtain the crude enzyme extract. Preparation of the reaction mixture encompassed a mixture of 100 mL of phosphate buffer (pH 6.0) in a beaker, and 58 μL of guaiacol (2-methoxyphenol) was added, heated, and stirred until dissolved. Thereafter, 56 μL of 30% H_2_O_2_ was added, mixed, and stored at 4 °C for subsequent use. An aliquot of 3 mL of reaction mixture was added to 0.2 mL of enzyme solution (buffer solution following proper dilution), and the absorbance at 470 nm was determined after 10 s and 120 s. PBS (pH 6.0) was employed instead of enzyme solution as a control. POD first collected bacteria or cells into a centrifuge tube and discarded the supernatant after centrifugation. According to the number of cells: the liquid volume (mL) was extracted at a ratio of 500–1000:1, and the cells were lysed using ultrasound; the work rate was 20% or 200 W, and the ultrasound pulse duration was 3 s, with an interval of 10 s, repeated 30 times. The sample was centrifuged at 8000× *g* at 4 °C for 10 min. The supernatant was obtained and incubated on ice for measurement. According to the tissue mass (g), the extract liquid volume (mL) was a 1:5 to 10 ratio operating using ice bath homogenization. Samples were centrifuged at 8000× *g* at 4 °C for 10 min. The supernatant was obtained and incubated on ice for measurement. A spectrophotometer was preheated for over 30 min, with the wavelength adjusted to 470 nm, and distilled water was used as a blank. The results were similar following replication in triplicate [56].

### 4.7. Transcriptome Sequencing and Data Analysis

RNA was extracted from treated cotton leaves. Libraries were constructed and sequenced following standard experimental procedures provided by Illumina 2000 systems. DESeq 2 was employed to identify differentially expressed genes (DEGs) as follows: |multiploid changes|≥ 1. The resulting *p*-values were adjusted using the Benjamini and Hochberg methods. Genes with an adjusted *p*-value < 0.05, as determined by DESeq 2, were designated as differentially expressed [57]. EggNOG-mapper v2 was used for gene annotation. The Genetic Ontology (GO) and Kyoto Encyclopedia of Genes and Genomes (KEGG) pathway enrichment analyses of DEGs were performed using the Cluster Analyzer R package [58].

### 4.8. Expression Analysis of Defense Marker Genes and Characterization of Plant Hormones

Leaf tissue from cotton plants infected with *V. dahliae* with silenced *GhIQD1* was frozen in liquid nitrogen for subsequent RNA extraction. Control plants were exposed to distilled water. SA pathway-related genes *PR5*, *NPR1*, and *NPR3* were detected. *NPR1* and *NPR3* were SA receptor-related genes [59], while *PR5* is often used as a marker for salicylic acid-mediated systemically acquired resistance (SAR) activation [60]. JA pathway-related genes *AOC*, *AOS*, and *PDF1.2* were also identified. Allene oxide cyclase (AOC) is a critical enzyme for JA biosynthesis [61], and allene oxide synthase (AOS) is the second enzyme utilized in the biosynthesis of the plant defensive hormone jasmonic acid (JA) [62]. The *PYR1/PYL/RCAR* protein operates as an ABA receptor that inhibits *PP2C* activation of *SnRK2*, causing phosphorylation of other effectors in the ABF and ABA response pathways [63]. All RT-qPCR assays were conducted in triplicate. SA, JA, and ABA levels were assessed in leaves 48 h after inoculation with TRV::00 and TRV::GhIQD1. Three biological replicates were established using high-performance liquid chromatography [64].

## 5. Conclusions

The protein encoded by the *GhIQD1* gene is a calmodulin binding protein, with a gene CDS length of 1314 bp and encoding 437 amino acids. It exhibits high conservation across various cotton varieties and other plants. *GhIQD1* has demonstrated its significance in cotton through VIGS silencing experiments, highlighting its importance in plant disease resistance. Silencing *GhIQD1* led to increased susceptibility of cotton to V. dahliae infection. Conversely, overexpression of *GhIQD1* in tobacco significantly enhanced resistance against V. Dahliae and reduced disease symptoms. Transcriptome analysis revealed the underlying mechanism of *GhIQD1* in plant disease resistance, showing that its overexpression promotes synthesis of photosynthetic substances, enriches amino acid metabolic pathways, activates secondary metabolic synthesis pathways, and triggers plant hormone reactions. Further research suggests that *GhIQD1* may influence pathogen resistance by regulating JA, ABA, and SA signaling pathways in plants. Its overexpression induces extensive changes within these signaling pathways, thereby enhancing disease resistance in plants. In summary, the regulatory role played by the *GhIQD1* gene is crucial for enhancing cotton’s pathogen resistance through participation in various signaling and metabolic pathways. These findings provide an important theoretical and scientific foundation for breeding resistant varieties and developing plant protection strategies.

## Data Availability

The data presented in this study are available upon request from the corresponding author.
